# Relations Between Dimensions of Emotional Intelligence, Specific Aspects of Empathy, and Non-verbal Sensitivity

**DOI:** 10.3389/fpsyg.2019.01066

**Published:** 2019-05-14

**Authors:** Enrique G. Fernández-Abascal, María Dolores Martín-Díaz

**Affiliations:** Department of Basic Psychology II, National University of Distance Education, Madrid, Spain

**Keywords:** trait emotional intelligence, empathy, affective empathy, cognitive empathy, non-verbal sensitivity, age differences, gender differences

## Abstract

In this work, on the one hand, we examined the relationship between emotional intelligence (EI) and empathy and, on the other, the relationship between EI and non-verbal sensitivity, through two independent studies. The first study analyzed the relationship between dimensions of EI and aspects of empathy, in a sample of 856 participants who completed two measures of EI, the trait meta-mood scale (TMMS) and the trait emotional intelligence questionnaire (TEIQue), and a measure of empathy, the interpersonal reactivity index (IRI). The results showed a similar pattern of significant positive relations in all the EI domains with empathic perspective-taking (PT), and significant negative relationships with empathic personal distress (PD), except for the EI domain of attention, which had a positive relationship. Stepwise multiple regression analyses showed that the dimension that best predicted empathic PT and empathic concern (EC) was the emotionality factor; whereas attention best predicted empathic fantasy; and the self-control factor best predicted PD, although negatively. Gender emerged as a predictor of three empathic aspects, fantasy, EC, and PD, with women obtaining higher scores than men in all of them. Age was the only predictor of fantasy, with a negative relationship. The second study involved 646 people who completed the same measures of EI as the participants of the first study and the mini-profile of non-verbal sensitivity (Mini-PONS). The results showed some significant relationships between EI dimensions and the channels and quadrants of the MiniPONS. Stepwise multiple regression analyses showed that very few EI dimensions predicted non-verbal sensitivity, with attention obtaining the best result. Both gender and age emerged as predictors, some in unique cases, of channels, quadrants, and of the total score of non-verbal sensitivity; age had a negative relationship, and women obtained higher scores than men.

## Introduction

Emotional intelligence (EI) is a multidimensional construct, “researchers refer to EI as a set of abilities or perceptions concerning the way individuals identify, make use of, deal with, and process emotions” (Andrei et al., 2016, p. 361).

The most widely recognized EI theoretical frameworks are the ability (Salovey and Mayer, 1990; Mayer and Salovey, 1997) and the trait models (Bar-On, 1997; Petrides and Furnham, 2001). The ability approaches which examine relatively discrete mental abilities that process emotional information (Mayer et al., 2008), and trait EI or trait emotional self-efficacy approaches where trait EI refers to a constellation of emotional self-perceptions located at the lower levels of personality hierarchies (Petrides et al., 2007a; see Zeidner et al., 2008, for a review of differences between the trait and ability EI frameworks). “Essentially, it concerns people’s perceptions of their emotional abilities, comprehensively encompassing the affective aspects of personality” (Andrei et al., 2016, p. 262).

Although EI has received different operationalizations, there is a broad consensus that it can be divided into two general streams: maximum performance tests, which assess actual levels of EI performance (ability EI), and self-report questionnaires, which reflect typical EI functioning (trait EI or emotional self-efficacy) (Pérez et al., 2005; Siegling et al., 2014; Fernández-Abascal and Martín-Díaz, 2015).

Andrei et al. (2016, p. 261) indicate that, “although distinct constructs, ability and trait EI are not mutually exclusive, and their bifurcation is now widely recognized within the scientific literature.”

“Although there is some divergence amongst EI researchers on the best model for EI, there is agreement that it covers the ways in which people differ in their emotional capabilities, both in the intrapersonal (mood regulation, stress management, and perceiving one’s own emotions) and interpersonal (social skills, perceiving others’ emotions) domains” (Austin et al., 2007, p. 685).

Empathy is a complex multidimensional concept (Davis, 1980). “There are probably nearly as many definitions of empathy as people working on the topic” (De Vignemont and Singer, 2006, p. 435), and a range of definitions used (e.g., see Batson, 2009, for a review of empathy definitions used). Empathy is broadly defined as the capacity to imagine, experience, and understand what another person is feeling (Gilet et al., 2013).

“The term empathy is used to refer to two related, yet different human abilities: mental perspective taking (cognitive empathy) and the vicarious sharing of emotion (affective empathy)” (Barlińska et al., 2018, p. 2).

In practice, empathy is defined by two major types, affective and cognitive, and is typically conceptualized as “a two-component model integrating both an affective and a cognitive dimension” (Braun et al., 2015, p. 736).

Empathy refers to a phenomenon that “requires both the ability to share the emotional experience of the other person (affective component) and an understanding of the other person’s experience (cognitive component)” (Decety and Jackson, 2004, p. 73).

“Affective empathy is the extent to which one feels what another person is feeling, and cognitive empathy is the extent to which one infers the thoughts, intentions, and feelings of another person” (Olderbak and Wilhelm, 2017, p. 1093).

Emotional intelligence and empathy are related but distinct constructs. There are similarities between some of their key concepts, “for example, Petrides and Furnham (2001) have identified the emotional management of others, emotion perception, relationship skills, social competence, and trait empathy as key components of EI” (Muncer and Ling, 2006, p. 1118). Austin et al. (2007) point out that empathy overlaps with interpersonal EI and covers the ability to be aware of and understand another person’s feelings.

From the perspective of Mayer and Salovey (1997), it is considered that emotionally intelligent people can not only better perceive, understand, and manage their own emotions, but are also more skilled at extrapolating these abilities of perception, understanding, and managing to the emotions of others.

Petrides et al. (2004) stated that most models of trait EI use aspects of affect-related functioning such as emotion awareness, empathy, and relationship skills to assess EI.

When examining the relationship between emotion perception and empathy Olderbak and Wilhelm (2017) point out that “there are three categories of theories positing a relation between emotion perception and empathy” (Olderbak and Wilhelm, 2017, p. 1094), and each one proposes a positive relation between the constructs (see Olderbak and Wilhelm, 2017, for a detailed description of the three categories of theories). “The first category proposes a within-subject process where emotion perception leads to affective and/or cognitive empathy…The second category includes theories that propose that emotion perception and empathy are lower-order factors of a single higher-order EI factor, implying a between-subject organization…The third category includes theories that propose emotion perception as a lower-order factor of EI and empathy, while related with emotion perception and its relevant higher-order constructs, is a construct distinct from EI” (Olderbak and Wilhelm, 2017, pp. 1094–1095).

The concept of non-verbal sensitivity is related to the concepts of EI and empathy. Riggio and Darioly (2016) point out that “Non-verbal sensitivity refers to the ability of individuals to read and decode non-verbal cues in others and, importantly, the ability to correctly interpret the meaning of those cues” (p. 589).

Non-verbal sensitivity toward others’ affective communication is a major part of EI. Bänziger et al. (2011a) indicate that “a central component of such competence is to correctly infer the complex affective states that individuals experience and communicate in specific social situations, which include fairly standardized communicative actions like requesting, commanding, scolding, pleading, etc.” (Bänziger et al., 2011a, p. 202).

In several models of EI, the accurate interpretation of non-verbal emotional information is considered to be a precondition for successfully understanding and managing others’ emotions, thus facilitating interpersonal communication and individual goal attainment (Schlegel et al., 2017).

Besides “recognizing emotion cues, both verbal and non-verbal, another important factor in empathic communication” (Blanch-Hartigan, 2011, p. 370). “It is assumed that non-verbal sensitivity is an important contributor to the concept of empathy, with the ability to read others’ emotions being critical for empathic understanding” (Riggio and Darioly, 2016, p. 589).

In the existing literature, many studies highlight the relationship among the constructs of EI, empathy, and non-verbal sensitivity to a greater or lesser extent but the investigations that have analyzed these relations have generally not differentiated between dimensions of the same construct. Also, in most cases, they have used a single score of either EI or empathy.

It is necessary therefore to analyze the relationship between the different dimensions of EI and the many aspects of non-verbal sensitivity and empathy, as well as the contribution of the EI dimensions to aspects of empathy and non-verbal sensitivity, leading to a better understanding of the interrelationships between these constructs.

### The Present Research

The present study was designed to provide more information about the relationship between EI and empathy, on the one hand, and EI and non-verbal sensitivity, on the other hand. The main objective is to define the specific dimensions of EI that best predict aspects of empathy and various aspects of non-verbal sensitivity.

Regarding the scales designed to evaluate EI, as indicated by Martins et al. (2010) in their meta-analysis and by Baudry et al. (2018) in their review, two types of measures co-exist, which represent the “narrow” and the “comprehensive” models.

“The measure of narrow models is generally based on the trait meta-mood scale (TMMS; Salovey et al., 1995) and the emotional intelligence scale (EIS; Schutte et al., 1998). These scales assess the perception of individuals about their current level of emotional competences” (Baudry et al., 2018, p. 207).

“The comprehensive models are examined by scales based on emotional competences as well as emotion-related personality traits (e.g., general mood, optimism, and well-being) with the emotional quotient inventory (EQ-I; Bar-On, 2006) and the trait emotional intelligence questionnaire (TEIQue; Petrides, 2009a). These scales assess the perception of individuals about their emotion-related personality characteristics or personal disposition (Baudry et al., 2018, p. 207).

As indicated by Martins et al. (2010), the TMMS and the TEIQue are two of the most frequently used measures.

The TMMS is based on Salovey and Mayer’s (1990) EI model. This self-report measure evaluates a “reasonable operationalization of aspects of EI” (Salovey et al., 1995, p. 147). This instrument provides an index of what researchers have called a proxy for perceived EI (PEI) (Salovey et al., 2002; Extremera and Fernández-Berrocal, 2005; Paek, 2006; Fernández-Berrocal and Extremera, 2008).

The TEIQue provides an operationalization for Petrides’s model (Petrides and Furnham, 2001; Petrides et al., 2007a). The construct seems to encompass two kinds of variance, a portion that is scattered across the higher-order dimensions of established personality taxonomies and a portion of variance that lies outside these dimensions (Petrides et al., 2007a). These two scales do not examine the same underlying dimensions of and mechanisms involved in EI (Baudry et al., 2018).

Several self-report measures have been developed to assess empathy (for a review, see Pedersen, 2009) but, currently, the interpersonal reactivity index (IRI), an individual difference measure of empathy based on such a multidimensional approach and developed by Davis (1980) is one of the most commonly used self-report questionnaires to assess empathic tendencies in adults (Gilet et al., 2013). “Rather than treating empathy as a simple unipolar construct, the rationale underlying the IRI is that empathy can best be considered as a set of constructs, related in that they all concern responsivity to others but are also clearly discriminable from each other” (Davis, 1983, p. 113). Davis (1980) instrument acquires special relevance and usefulness to assess empathy from a multidimensional perspective that includes both cognitive and emotional factors. The most outstanding feature of this instrument is that it measures both the cognitive aspect and the individual’s emotional reaction when adopting an empathetic attitude.

Some tests that collect static and video-taped visual stimuli have been developed to evaluate non-verbal emotional sensitivity, as well as prosodic tests. One of the most widely used and best known measures of the individual capacity to decode non-verbal behavior is the profile of non-verbal sensitivity (PONS) of Rosenthal et al. (1979), which was developed for assessing individual differences in the ability to recognize emotions and interpersonal attitudes, and to communicate intentions through various non-verbal expressive channels.

Bänziger et al. (2011a) developed a reduced version of the mini profile of non-verbal sensitivity (MiniPONS), which retains most of the non-verbal expressive channels (and their combination) of the original test in order to gather the broad variety of skills to decode emotional expression.

Taking into account that EI includes intrapersonal and interpersonal domains, and that the measures usually evaluate a number of subcomponents of EI, as well as combining them into an overall score, for our two studies, we selected the two most commonly used measures of EI that assess different EI domains and thus encompass the greatest possible number of EI domains. These measures are the TMMS and the TEIQue.

The measure of empathy selected for the present study was the IRI, and to evaluate non-verbal sensitivity in the second study, we selected mini profile of non-verbal sensitivity (MiniPONS).

This work has two main objectives. In the first study, the goal was to determine the dimensions of EI that predict different aspects of empathy; in the second study, to determine the EI dimensions that predict various aspects of non-verbal sensitivity.

In the first study, we hypothesized that only a few dimensions of EI would predict the evaluated aspects of empathy. However, in the second study we hypothesized that the few dimensions of EI that predict aspects of non-verbal sensitivity will be different from the predictive dimensions of the aspects of empathy evaluated in the first study.

## Study 1

### Materials and Methods

#### Participants and Procedure

Participants were 856 undergraduate students, with a mean age of 33.62 years (*SD* = 10.46), and age ranging between 18 and 67 years. There were 185 (21.6%) men in the sample, mean age 36.96 years (*SD* = 11.17), age range between 18 and 67 years, and 671 (78.4%) women, mean age 32.70 years (*SD* = 10.07), age range between 18 and 62 years.

These people were recruited in the National University of Distance Education (UNED), and volunteered to take part in this study. They were not rewarded for taking part in the study. Due to the characteristics of the National University of Distance Education (UNED), the participants are representative of the general population, they study and work, practice different professions, live in urban and rural environments, and have a very wide age range.

All subjects gave written informed consent to participate in the study. This study followed the Declaration of Helsinki and ethical guidelines. The study protocol was approved by the Bioethics Committee of the Faculty of Psychology of the National University of Distance Education.

#### Measures

##### Trait EI measures

###### Trait meta-mood scale (TMMS; Salovey et al., 1995)

The TMMS was designed to assess the way people reflect on their moods, and thus, it was conceived as an index of perceived EI (Salovey et al., 2002). The TMMS “has been widely used as a measure for assessing stable individual differences in people’s beliefs to attend to, discriminate and regulate their moods, and emotions” (Extremera et al., 2011, p. 510).

The scale has three factors that provide three subscale scores: attention or attention to feelings, clarity or clarity of feelings, and repair or mood repair.

Attention or attention to feelings, evaluates the amount of attention paid to one’s emotional states, perceived ability to attend to moods and emotions. clarity or clarity of feelings, refers to understanding one’s emotional states, perceived ability to understand and discriminate between different moods and emotions. Repair or Mood Repair, relating to the ability to regulate one’s emotional states, perceived ability to maintain positive, and repair negative moods and emotions (Salovey et al., 1995).

We used the validated Spanish shorter version of the TMMS (see Fernández-Berrocal et al., 2004), which includes 24 items from the original version, this version has shown aceptable internal consistency and satisfactory test–retest reliability (Fernández-Berrocal et al., 2004).

The final Spanish version consists of three subscales with 8 items for each subscale. Participants rate the extent to which they agree with each item on a 5-point Likert-type scale ranging from 1 (*Strongly disagree*) to 5 (*Strongly agree*).

###### Trait emotional intelligence questionnaire (TEIQue; Petrides and Furnham, 2003; Petrides, 2009a,b)

The TEIQue comprehensively covers the sampling domain of trait EI (Petrides et al., 2011). We used the most recent version of this questionnaire, the TEIQue v 1.50 (Petrides, 2009b). This version consists of 153 items (rated on a 7-point Likert scale) and encompasses 13 facets, organized in four factors: well-being, self-control, emotionality, and sociability. Two additional facets (adaptability and self-motivation) contribute directly to the Global Trait EI score.

The well-being factor, pertaining to dispositional mood, refers to a generalized sense of well-being extending from past achievements to future expectations. The emotionality factor, reflects the ability to identify and express feelings and emotions, and to use these faculties to maintain close relationships with significant others. The sociability factor, interpersonal utilization and management of emotions, regarding the capacity to assert oneself as well as to influence others’ emotions and decisions. The self-control factor, concerning the ability to regulate one’s impulses and emotions, as well as to manage external pressures and stress (Petrides and Furnham, 2003; Petrides, 2009b).

The well-being factor includes the facets of self-esteem, trait happiness, and trait optimism. The emotionality factor includes the facets of emotion perception (self and others), emotion expression, trait empathy, and relationships. The sociability factor includes the facets of social awareness, emotion management (others), and assertiveness. The self-control factor includes the facets of emotion regulation, stress management, and impulsiveness (low).

In the 1.50 version of the TEIQue, participants rate their degree of agreement with each item on a 7-point Likert-type scale ranging from 1 (*Completely disagree*) to 7 (*Completely agree*).

The instrument has shown excellent psychometric properties in several studies (Mikolajczak et al., 2007; Freudenthaler et al., 2008; Jolic-Marjanovic and Altaras-Dimitrijevic, 2014). “The solid psychometric basis of the TEIQue instruments is reflected in the cross-cultural stability of its four-factor structure, which has been replicated in several languages” (see Andrei et al., 2016, p. 262).

##### Empathy measure

###### Interpersonal reactivity index (IRI, Davis, 1980, 1983)

This index is based on a multidimensional approach. “The rationale underlying the IRI is that empathy can best be considered as a set of constructs, related in that they all concern responsivity to others but are also clearly discriminable from each other” (Davis, 1983, p. 113). The IRI “acknowledges that empathy is composed of separate but related cognitive and affective components” (Young Kaelber and Schwartz, 2014, p. 279). The IRI contains 28 total items, with two cognitive and two emotional scales. Items are presented in randomized order using a 5-point Likert-type scale ranging from 0 (*Does not describe me*) to 4 (*Describes me very well*).

The two cognitive scales are perspective-taking (PT), which assesses the tendency to adopt the point of view of other people in everyday life, the ability to understand another person’s point of view; and fantasy (FS), which assesses the tendency to transpose oneself into the feelings and actions of fictitious characters in books, movies, and plays; that is, the person’s imaginative capacity to place him- or herself in fictitious situations. The two emotional scales are empathic concern (EC), which assesses the tendency to experience feelings of warmth, compassion, and concern for other people; and personal distress (PD), which assesses typical emotional reactions, but rather than other-oriented feelings of concern, it taps one’s own feelings of personal unease and discomfort in reaction to the emotions of others (Davis, 1983), measuring feelings of fear, apprehension, and discomfort at witnessing the negative experiences of others.

Each scale contains 7 items that are summed to create a total score for each scale which ranges from 0 to 28 points, with higher scores indicating greater empathy.

The IRI has good psychometric properties (Davis, 1980, 1983) and has been widely used in a variety of populations and validated in several languages including Chinese (Siu and Shek, 2005; Chiang et al., 2014), Dutch (De Corte et al., 2007), French (Gilet et al., 2013), German (Paulus, 2009), Italian (Sartori and Meneghini, 2007), Spanish (Pérez-Albéniz et al., 2003; Mestre et al., 2004), and Swedish (Cliffordson, 2002).

##### Statistical analysis

For all data analyses, we used the IBM SPSS Statistics for Windows, version 25.0 (IBM Corp. Released, 2017). For all continuous variables, correlations were reported as Pearson product moment correlations (two-tailed).

In order to analyze the differences between men and women, we used Student’s paired-sample *t*-test for independent samples. When the variances were not significantly different (probability of *F* > 0.05), Student’s *t*-test was used with pooled variances; and when they were significantly different, it was used with separate variances.

To explore the predictive value of the EI dimensions as the independent variables, stepwise multiple regression analyses were performed, with aspects of empathy as the dependent variable.

To integrate the results of the stepwise multiple regression analyses, we examined a model of relationships between variables. The program used was Amos (version 25.0).

## Results

### Internal Consistencies and Descriptive Statistics

Cronbach alphas, means and standard deviations were calculated for each scale. Table 1 presents the results for the total sample of participants and by gender. All the internal consistency values were within acceptable levels.

**Table 1:** Cronbach’s alphas, means, and standard deviations of the variables examined.

Trait meta-mood scale (TMMS)							
	**Total Sample *N* = 856**			**Male *N* = 185**		**Female *N* = 671**	
			
**Scale (number of items)**	**Cronbach alpha**	***M***	***SD***	***M***	***SD***	***M***	***SD***

Attention (8)	0.887	27.55	6.59	25.14	6.56	28.22	6.44
Clarity (8)	0.899	29.01	6.77	28.40	6.92	29.18	6.73
Repair (8)	0.876	27.78	6.59	27.72	6.51	27.79	6.62

**Trait emotional intelligence questionnaire (TEIQue)**							

	**Total Sample *N* = 856**			**Male *N* = 185**		**Female *N* = 671**	
			
**Scale (facet) (number of items)**	**Cronbach alpha**	***M***	***SD***	***M***	***SD***	***M***	***SD***

Emotion expression (10)	0.907	4.70	1.37	4.41	1.31	4.78	1.38
Empathy (trait empathy) (9)	0.767	5.55	0.79	5.37	0.83	5.60	0.77
Self-motivation (10)	0.813	5.14	0.95	5.03	0.96	5.18	0.94
Emotion regulation (12)	0.839	4.42	0.96	4.68	0.98	4.34	0.94
Happiness (trait Happiness) (8)	0.885	5.52	1.13	5.50	1.19	5.53	1.11
Social awareness (11)	0.844	4.92	1.00	4.88	1.05	4.93	0.99
Impulsiveness (low) (9)	0.782	4.97	0.96	5.06	0.95	4.94	0.96
Emotion perception (self an others) (10)	0.815	5.11	0.94	4.91	0.95	5.17	0.93
Self-esteem (11)	0.855	4.86	1.02	4.96	0.99	4.84	1.03
Assertiveness (9)	0.742	4.72	0.92	4.77	0.92	4.70	0.92
Emotion management (others) (9)	0.803	4.90	0.98	4.94	0.99	4.89	0.97
Optimism (trait optimism) (8)	0.906	5.28	1.20	5.34	1.18	5.26	1.20
Relationship (9)	0.627	5.59	0.74	5.42	0.78	5.64	0.72
Adaptability (9)	0.782	4.72	0.94	4.79	0.98	4.71	0.92
Stress management (10)	0.839	4.55	1.10	4.88	1.12	4.45	1.07
Well-being factor (3 facets)	0.877	5.22	1.00	5.26	1.01	5.21	1.00
Emotionality factor (4 facets)	0.748	5.24	0.75	5.03	0.76	5.30	0.73
Sociability factor (3 facets)	0.772	4.85	0.80	4.86	0.84	4.84	0.79
Self-control factor (3 facets)	0.840	4.64	0.88	4.87	0.89	4.58	0.86
Global trait EI (15 facets)	0.910	5.00	0.67	5.00	0.73	5.00	0.65

**Interpersonal reactivity index (IRI)**							

	**Total Sample *N* = 856**			**Male *N* = 185**		**Female *N* = 671**	
			
**Scale (number of items)**	**Cronbach alpha**	***M***	***SD***	***M***	***SD***	***M***	***SD***

Perspective-taking (7)	0.805	18.54	5.10	17.92	4.95	18.71	5.13
Fantasy (7)	0.830	18.21	6.02	15.59	5.88	18.94	5.86
Empathic concern (7)	0.684	21.80	4.12	19.58	4.32	22.41	3.85
Personal distress (7)	0.753	10.17	5.07	8.71	5.32	10.58	4.93


### Correlations Between EI Dimensions and Empathic Interpersonal Reactivity Index (IRI)

To test the relationship between the EI dimensions and this aspect of empathy, Pearson product moment correlations were computed between the dimensions of the EI measures (TMMS, TEIQue) and the IRI. We also examined the relation between participants’ age and the IRI.

As shown in Table 2, almost all the EI dimensions of the TMMS had a significant and positive correlation with the aspects of the IRI, except for the EI dimensions clarity and repair, which had significant but negative correlations with PD, and repair, which had no significant relationship with fantasy.

**Table 2:** Pearson correlations between the emotional intelligence dimensions and the Interpersonal Reactivity Index.

Interpersonal reactivity index (IRI)				
	**Perspective-taking**	**Fantasy**	**Empathic concern**	**Personal distress**

**Trait meta-mood scale (TMMS)**				
Attention	0.180^∗∗∗^ (*p* = 0.000)	0.356^∗∗∗^ (*p* = 0.000)	0.285^∗∗∗^ (*p* = 0.000)	0.194^∗∗∗^ (*p* = 0.000)
Clarity	0.293^∗∗∗^ (*p* = 0.000)	0.081^∗^ (*p* = 0.018)	0.120^∗∗∗^ (*p* = 0.000)	-0.316^∗∗∗^ (*p* = 0.000)
Repair	0.386^∗∗∗^ (*p* = 0.000)	0.011 (*p* = 0.759)	0.118^∗∗∗^ (*p* = 0.001)	-0.379^∗∗∗^ (*p* = 0.000)
**Trait emotional intelligence questionnaire (TEIQue)**				
Well-being factor	0.227^∗∗∗^ (*p* = 0.000)	-0.120^∗∗∗^ (*p* = 0.000)	0.044 (*p* = 0.200)	-0.443^∗∗∗^ (*p* = 0.000)
Emotionality factor	0.446^∗∗∗^ (*p* = 0.000)	0.069^∗^ (*p* = 0.044)	0.323^∗∗∗^ (*p* = 0.000)	-0.343^∗∗∗^ (*p* = 0.000)
Sociability factor	0.206^∗∗∗^ (*p* = 0.000)	0.042 (*p* = 0.215)	0.166^∗∗∗^ (*p* = 0.000)	-0.452^∗∗∗^ (*p* = 0.000)
Self-control factor	0.322^∗∗∗^ (*p* = 0.000)	-0.289^∗∗∗^ (*p* = 0.000)	-0.130^∗∗∗^ (*p* = 0.000)	-0.569^∗∗∗^ (*p* = 0.000)
Global trait EI	0.391^∗∗∗^ (*p* = 0.000)	-0.106^∗∗^ (*p* = 0.002)	0.133^∗∗∗^ (*p* = 0.000)	-0.571^∗∗∗^ (*p* = 0.000)
**Age**	0. 024 (*p* = 0.478)	-0.259^∗∗∗^ (*p* = 0.000)	0.038 (*p* = 0.261)	-0.199^∗∗∗^ (*p* = 0.000)
**Interpersonal reactivity index (IRI)**				
Perspective-taking		0.174^∗∗∗^ (*p* = 0.000)	0.351^∗∗∗^ (*p* = 0.000)	-0.168^∗∗∗^ (*p* = 0.000)
Fantasy			0.378^∗∗∗^ (*p* = 0.000)	0.249^∗∗∗^ (*p* = 0.000)
Empathic concern				0.100^∗∗^ (*p* = 0.003)


*Total Sample *N* = 856. ^∗∗∗^*p* ≤ 0.001 (two tailed); ^∗∗^*p* ≤ 0.01 (two tailed); ^∗^*p* ≤ 0.05 (two tailed).*

All the factors and the total EI score of the TEIQue had a significant and positive relationship with empathic PT and a significant and negative relationship with empathic PD. In contrast to these aspects, the relationships with the other two aspects of empathy, fantasy and EC, were not homogeneous. The well-being and self-control factors and global trait EI had significant negative relationships with fantasy, whereas the emotionality factor had a significant and positive relationship with fantasy. The sociability factor had no significant relationship with fantasy. The emotionality factor, the sociability factor, and global trait EI presented significant and positive relationships with EC, and the self-control factor had a significant and negative relationship with EC, whereas the well-being factor did not present a significant relationship with EC.

Age presented significant and negative relationships with fantasy and PD.

The relationships between the four aspects of empathy evaluated with the IRI were significant and positive, except for the relationship of PT with PD, which was negative.

### Stepwise Multiple Regression With EI Dimensions as Predictor Variables and Each One of the Empathic Aspects of the IRI as Criterial Variables

Prior to the stepwise multiple regression analysis, the relationships between independent variables (TMMS, TEIQue) and dependent variables (IRI) were examined. EI dimensions variables significantly associated with empathic aspects variables were considered candidate predictors and entered into the stepwise multiple regression analysis. To avoid the collinearity problem with the TEIQue factors, we did not enter the general EI measure from this questionnaire in any analysis.

With the independent variable age, we followed the same procedure as with the EI dimensions, considering age an independent variable in the situations in which it had a significant relation with some aspect of empathy.

Gender was entered as an independent variable to determine whether it predicted empathy only in the cases in which significant differences between men and women were found, that is, all the empathy variables except for PT, where no significant mean differences were found, *t* = -1858 (854), *p* = 0.064, *d* = -0.157. Gender differences were observed in fantasy, *t*(854) = -6.871, *p* = 0.000, *d* = -0.571, EC, *t*(854) = -8.598, *p* = 0.000, *d* = -0.691, and PD, *t*(854) = -4.480, *p* = 0.000, *d* = -0.364, with a higher mean score in women than in men in these three aspects of empathy (see Table 2).

The results are presented in Table 3. In general, not all the dimensions of EI did have the same predictive power for empathy.

**Table 3:** Stepwise multiple regression analysis.

Dependent variables: Components of the interpersonal reactivity index (IRI)										
Independent variables: Dimensions of the trait meta-mood scale (TMMS) and trait emotional intelligence questionnaire (TEIQue)										
**Model**	***R***	***R*^2^**	***R*^2^ adjusted**	***R*^2^ change**	***F*(df)**	***p***	**ß**	**ß standarized**	***t***	***p***

**Dependent variable: Perspective-taking**										
Model 1: Emotionality factor	0.446	0.199	0.198	0.199	212.156 (1,854)^∗∗∗^	0.000	3.030	0.446	14.566^***^	0.000
Model 2: Emotionality factor							2.347	0.346	10.596^***^	0.000
Repair	0.498	0.248	0.246	0.049	140.604 (2,853)^∗∗∗^	0.000	0.188	0.243	7.451^***^	0.000
Model 3: Emotionality Factor							2.988	0.440	12.310^***^	0.000
Repair							0.274	0.354	9.549^***^	0.000
Well-being factor	0.527	0.278	0.275	0.030	109.142 (3,852)^∗∗∗^	0.000	-1.241	-0.244	-5.917^***^	0.000
Model 4: Emotionality factor							2.811	0.414	11.479^***^	0.000
Repair							0.253	0.327	8.740^***^	0.000
Well-being factor							-1.479	-0.291	-6.830^***^	0.000
Self-control factor	0.539	0.291	0.287	0.013	87.155 (4,851)^∗∗∗^	0.000	0.823	0.142	3.948^***^	0.000
Model 5: Emotionality factor							2.466	0.363	9.423^***^	0.000
Repair							0.234	0.302	7.993^***^	0.000
Well-being factor							-1.334	-0.263	-6.094^***^	0.000
Self-control factor							1.098	0.189	4.968^***^	0.000
Attention	0.549	0.301	0.297	0.010	73.213 (5,850)^∗∗∗^	0.000	0.090	0.116	3.559^***^	0.000
Model 6: Emotionality factor							2.986	0.440	9.884^***^	0.000
Repair							0.250	0.323	8.480^***^	0.000
Well-being factor							-1.385	-0.273	-6.351^***^	0.000
Self-control factor							1.205	0.208	5.429^***^	0.000
Attention							0.104	0.134	4.082^***^	0.000
Clarity	0.557	0.310	0.305	0.009	63.670 (6,849)^∗∗∗^	0.000	-0.102	-0.136	-3.384^***^	0.001
Model 7: Emotionality factor							3.260	0.480	10.215^***^	0.000
Repair							0.251	0.325	8.553^***^	0.000
Well-being factor							-1.237	-0.244	-5.505^***^	0.000
Self-control factor							1.178	0.203	5.319^***^	0.000
Attention							0.104	0.135	4.125^***^	0.000
Clarity							-0.098	-0.130	-3.258^***^	0.001
Sociability factor	0.562	0.316	0.310	0.005	55.898 (7,848)^∗∗∗^	0.000	-0.617	-0.097	-2.589^**^	0.010
**Dependent variable: Fantasy**										
Model 1: Attention	0.356	0.126	0.125	0.126	123.667 (1,854)^∗∗∗^	0.000	0.325	0.356	11.121^***^	0.000
Model 2: Attention							0.277	0.303	9.403^***^	0.000
Self-control factor	0.412	0.169	0.168	0.043	87.026 (2,853)^∗∗∗^	0.000	-01.463	-0.214	-6.644^***^	0.000
Model 3: Attention							0.261	0.286	8.969^***^	0.000
Self-control factor							-1.191	-0.174	-5.344^***^	0.000
Age	0.442	0.196	0.193	0.026	69.119 (3,852)^∗∗∗^	0.000	-0.097	-0.169	-5.275^***^	0.000
Model 4: Attention							0.211	0.231	6.833^***^	0.000
Self-control factor							-1.774	-0.259	-6.913^***^	0.000
Age							-0.101	-0.176	-5.551^***^	0.000
Emotionality factor	0.463	0.214	0.210	0.018	57.908 (4,851)^∗∗∗^	0.000	1.302	0.162	4.441^***^	0.000
Model 5: Attention							0.204	0.223	6.611^***^	0.000
Self-control factor							-1.637	-0.239	-6.338^***^	0.000
Age							-0.093	-0.161	-5.080^***^	0.000
Emotionality factor							1.102	0.138	3.706^***^	0.000
Gender	0.473	0.224	0.219	0.010	49.089 (5,850)^∗∗∗^	0.000	1.548	0.106	3.327^***^	0.001
Model 6: Attention							0.190	0.208	6.049^***^	0.000
Self-control factor							-1.736	-0.254	-6.634^***^	0.000
Age							-0.095	-0.165	-5.193^***^	0.000
Emotionality factor							0.683	0.085	1.932	0.054
Gender							1.603	0.110	3.449^***^	0.001
Clarity	0.478	0.228	0.223	0.004	41.890 (6,849)^∗∗∗^	0.000	0.082	0.092	2.190^*^	0.029
**Dependent variable: Empathic concern**										
Model 1: Emotionality factor	0.323	0.104	0.103	0.104	99.468 (1,854)^∗∗∗^	0.000	1.773	0.323	9.973^***^	0.000
Model 2: Emotionality factor							2.636	0.480	13.988^***^	0.000
Self-control factor	0.448	0.200	0.198	0.096	106.872 (2,853)^∗∗∗^	0.000	-1.629	-0.348	-10.122^***^	0.000
Model 3: Emotionality factor							2.380	0.434	12.489^***^	0.000
Self-control factor							-1.417	-0.302	-8.721^***^	0.000
Gender	0.479	0.229	0.227	0.029	84.505 (3,852)^∗∗∗^	0.000	1.771	0.177	5.657^***^	0.000
Model 4: Emotionality factor							2.155	0.393	10.718^***^	0.000
Self-control factor							-1.207	-0.258	-6.970^***^	0.000
Gender							1.679	0.168	5.374^***^	0.000
Attention	0.489	0.239	0.236	0.010	66.908 (4,851)^∗∗∗^	0.000	0.069	0.111	3.333^***^	0.001
Model 5: Emotionality factor							2.622	0.478	11.004^***^	0.000
Self-control factor							-1.094	-0.234	-6.257^***^	0.000
Gender							1.611	0.161	5.180^***^	0.000
Attention							0.084	0.135	3.998^***^	0.000
Clarity	0.501	0.251	0.246	0.011	56.851 (5,850)^∗∗∗^	0.000	-0.090	-0.148	-3.590^***^	0.002
Model 6: Emotionality factor							2.550	0.465	10.725^***^	0.000
Self-control factor							-1.305	-0.278	-7.069^***^	0.000
Gender							1.592	0.159	5.150^***^	0.000
Attention							0.078	0.125	3.724^***^	0.000
Clarity							-0.103	-0.169	-4.067^***^	0.000
Repair	0.511	0.261	0.255	0.010	49.879 (6,849)^∗∗∗^	0.000	0.075	0.119	3.392^***^	0.001
**Dependent variable: Personal distress**										
Model 1: Self-control factor	0.569	0.324	0.323	0.324	409.195 (1,854)^∗∗∗^	0.000	-3.283	-0.569	-20.229^***^	0.000
Model 2: Self-control factor							-2.717	-0.471	-16.775^***^	0.000
Sociability factor	0.634	0.402	0.400	0.078	286.153 (2,853)^∗∗∗^	0.000	-1.859	-0.295	-10.517^***^	0.000
Model 3: Self-control factor							-2.511	-0.435	-14.951^***^	0.000
Sociability factor							-1.999	-0.318	-11.213^***^	0.000
Attention	0.643	0.413	0.411	0.012	200.222 (3,852)^∗∗∗^	0.000	0.089	0.115	4.168^***^	0.000
Model 4: Self-control factor							-2.294	-0.398	-12.446^***^	0.000
Sociability factor							-1.881	-0.299	-10.299^***^	0.000
Attention							0.098	0.127	4.566^***^	0.000
Repair	0.647	0.419	0.416	0.005	153.323 (4,851)^∗∗∗^	0.000	-0.067	-0.087	-2.796^**^	0.005
Model 5: Self-control factor							-2.243	-0.389	-12.153^***^	0.000
Sociability factor							-1.878	-0.298	-10.323^***^	0.000
Attention							0.089	0.115	4.110^***^	0.000
Repair							-0.070	-0.091	-2.935^**^	0.003
Gender	0.651	0.424	0.421	0.005	125.122 (5,850)^∗∗∗^	0.000	0.906	0.073	2.753^**^	0.006


*Total sample *N* = 856. ^∗∗∗^*p* ≤ 0.001; ^∗∗^*p* ≤ 0.01; ^∗^*p* ≤ 0.05.*

Regarding empathic PT, the prediction model contained seven predictors and was reached in seven steps, *F*(7,848) = 55.898*, p <* 0.001, accounting for 31.6% of the variance of PT (*R*^2^ = 0.316). The significant predictors of this model were the emotionality factor (*R*^2^ = 0.199), repair (*R*^2^ = 0.049), the well-being factor (*R*^2^ = 0.03), the self-control factor (*R*^2^ = 0.013), attention (*R*^2^ = 0.01), clarity (*R*^2^ = 0.009), and the sociability factor (*R*^2^ = 0.005). As can be seen, the EI dimension that best predicts empathic PT is the emotionality factor, which is made up of the following EI facets: Emotion Perception (self and others), emotion expression, trait empathy, and relationships.

In empathic fantasy, the prediction model contained six predictors and was reached in six steps, *F*(6,849) = 41.890, *p* < 0.001, accounting for 22.8% of the variance of the fantasy (*R*^2^ = 0.228). The significant predictors of this model were attention (*R*^2^ = 0.126), the self-control factor (*R*^2^ = 0.043; with a negative relation, see Table 2), age (*R*^2^ = 0.026; with a negative relation, see Table 2), the emotionality factor (*R*^2^ = 0.018), gender (*R*^2^ = 0.01; with the women obtaining higher scores than the men in this empathic aspect, see Table 1), and clarity (*R*^2^ = 0.004). The EI dimension that best predicted empathic fantasy was attention.

The prediction model of EC contained six predictors and was reached in six steps, *F*(6,849) = 49.879, *p* < 0.001, accounting for 26.1% of the variance (*R*^2^ = 0.261). The significant predictors of this model were the emotionality factor (*R*^2^ = 0.104), the self-control factor (*R*^2^ = 0.096; with a negative relation, see Table 2), gender (*R*^2^ = 0.029; with women obtaining higher scores than men, see Table 1), attention (*R*^2^ = 0.01), clarity (*R*^2^ = 0.011), and repair (*R*^2^ = 0.01). The emotionality factor was the EI dimension that best predicted EC, as was found with empathic PT.

Finally, in empathic PD, the prediction model contained five predictors and was reached in five steps, *F*(5,850) = 125.122, *p* < 0.001, accounting for 42.4% of the variance (*R*^2^ = 0.424). The significant predictors of this model were the self-control factor (*R*^2^ = 0.324; with a negative relation, see Table 2), the Sociability Factor (*R*^2^ = 0.078; with a negative relation, see Table 2), Atttention (*R*^2^ = 0.012), repair (*R*^2^ = 0.005; with a negative relation, see Table 2), and gender (*R*^2^ = 0.005; with women obtaining higher scores than men, see Table 1). The EI dimension that best predicted empathic PD was the self-control factor, which is made up of the following EI facets: emotion regulation, stress management, and impulsiveness (low). However, the relationship between PD and the Self-Control Factor was negative, as shown in Table 2. The relationship of the EI dimensions with empathic PD were all significant and negative, except for the EI dimension of attention, which was positive (see Table 2).

In particular, the EI dimensions that more strongly predicted aspects of empathy were the emotionality factor, which predicted PT and EC; the Self-Control Factor, which predicted PD; and attention, which predicted fantasy.

Based on the results obtained in the exploratory analyses carried out with correlations and regressions, we examined a model of relationships between the variables, using path analysis. The model included the predictors that were significant in each stepwise regression as independent or exogenous variables, and the aspects of empathy as dependent or endogenous variables.

To estimate the parameters, the maximum likelihood (ML) procedure was used. To determine possible univariate normality, the distribution of each variable with the skewness and kurtosis indexes (Kline, 2005) was examined, finding that all the variables presented a normal distribution. The bivariate correlations between the independent variables were also examined and, as no correlation exceeded the score of 0.85, there was no multicollinearity (Kline, 2005). There were no significant relationships between any of the independent variables.

The first model presented an acceptable fit, data fit, χ^2^_(18,*N* = 856)_ = 218.79, *p <* 0.0000, χ^2^/df = 12.15, CFI = 0.950, GFI = 0.961, RMSEA = 0.114. Although the value of χ^2^ did not indicate a good fit, we cannot consider this index because it is not a reliable indicator of the fit of a model with large samples (*N* > 200), as indicated by Hair et al. (2010).

We respecified the first model, eliminating from the analysis the non-significant relationships between the independent variables, and the second model presented a better fit, data fit χ^2^_(24,*_N_* = 856)_ = 225.63, *p <* 0.000, χ^2^/df = 9.40, CFI = 0.950, GFI = 0.960, RMSEA = 0.099.

**FIGURE 1:**
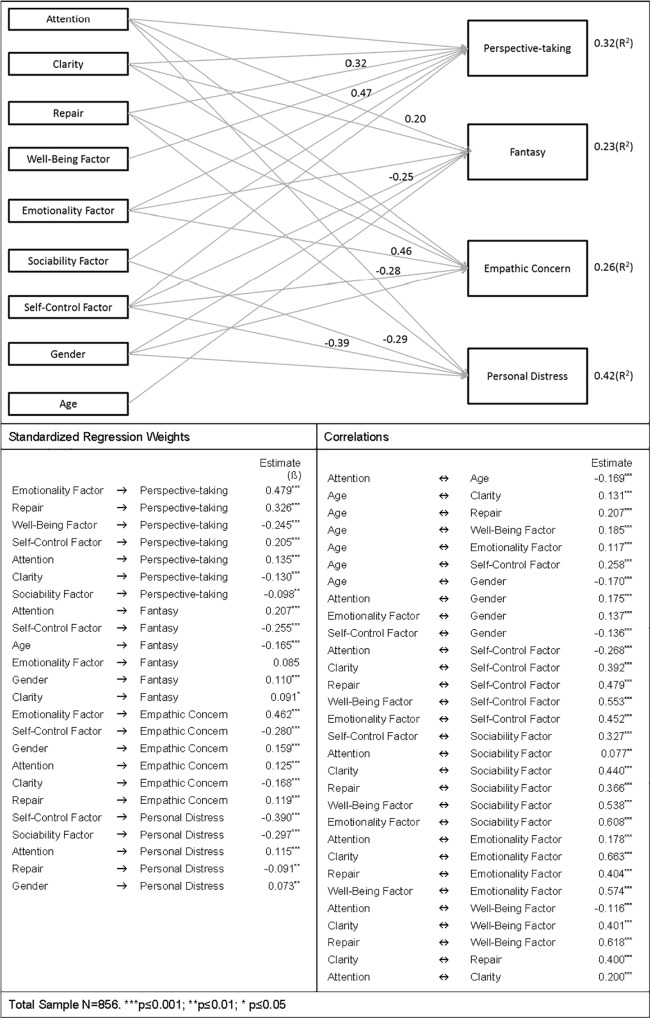
Model indicating standardized solutions and the most relevant relations.

Figure 1 presents the model, indicating standardized solutions and the most relevant relations. All the relations are presented in the tables included in that figure. The results show that the percentages of variance of each of the aspects of empathy explained by the independent variables are similar to those obtained in the regression analyses.

## Study 2

### Materials and Methods

#### Participants and Procedure

The participants in this second study were similar to those of the first study in their origin and characteristics, and were recruited in the same way, following the same procedure as in the first study. The sample comprised 646 undergraduate students with a mean age of 33.91 years (*SD* = 9.51), and age range from 18 to 63 years, who volunteered to take part in this study. There were 147 (22.8%) men in the sample, mean age 35.82 years (*SD* = 9.88), age range from 19 to 60 years; and 499 (77.2%) women, mean age 33.34 years (*SD* = 9.34), age range from 18 to 63 years.

All subjects gave written informed consent to participate in the study. This study followed the Declaration of Helsinki and ethical guidelines. The study protocol was approved by the Bioethics Committee of the Faculty of Psychology of the National University of Distance Education.

#### Measures

##### Trait EI measures

To assess EI, we applied the same scale and questionnaire as used in the first study, TMMS and TEIQue.

##### Non-verbal sensitivity measure

###### Mini profile of non-verbal sensitivity (MiniPONS; Bänziger et al., 2011a,b)

The MiniPONS is a short, multichannel version of the established profile of non-verbal sensitivity (PONS; Rosenthal et al., 1979). This test measures people’s ability to recognize the communication of feelings, attitudes, and intentions from non-verbal expressions in faces, voice, gestures, and body postures. The MiniPONS contains 64 video items from the original test, depicting a young woman in 20 different interpersonal situations varying widely with regard to their emotional quality. The recordings are shown in three different forms: videos with sound (16 Face-Voice video), videos without sound (16 face videos and 16 body videos), and audio stimuli (16 audio clips). In each audio recording, the woman produces an utterance that has been filtered to mask the verbal content.

After each presentation, two alternative situations are shown on the screen, describing what the young woman felt or wanted to communicate. The participants’ task is to decide which of the two alternatives best corresponds to her respective expression. “The scenarios include both positive and negative emotion and both dominant and submissive demeanor” (Roter et al., 2008, p. 400). Each scene lasts for 2 s, and the entire administration of the test requires approximately 15 min.

The scores obtained from the videos of the MiniPONS belong to the following channels:

Channel RS voice modality audio (randomized splicing) (8 videos).

Channel CF voice modality audio (content filtered) (8 videos).

Channel body modality video (16 videos).

Channel face modality video (16 videos).

Channel face+RS modality both audio and video (8 videos).

Channel face+CF modality both audio and video (8 videos).

Total MiniPONS (64 videos).

The scores can also be obtained that are relevant to the following quadrant design, combining valence and dominance: Negative dominant (16 videos), negative submissive (16 videos), positive dominant (16 videos), and positive submissive (16 videos).

In our work, we used the scores concerning the following channels:

Channel RS voice modality audio (randomized splicing) (8 videos) + Channel CF voice modality audio (content filtered) (8 videos), obtaining a single score with the sum of the two channels (16 videos).

Channel body modality video (16 videos).

Channel face modality video (16 videos).

Channel body modality video (16 videos) + Channel face modality video (16 videos), adding the scores obtained on both channels and obtaining a new score (32 videos).

Channel Face+RS modality both, audio and video (8 videos) + Channel face+CF modality both, audio and video (8 videos), obtaining a single score with the sum of the two channels (16 videos).

Total MiniPONS (64 videos).

Negative dominant (16 videos), negative submissive (16 videos), positive dominant (16 videos), and positive submissive (16 videos).

We obtained some scores that were not contemplated in the original MiniPONS, by performing more combinations of valence and dominance, as did Martínez-Sánchez et al. (2013), denominating them as follows:

Dominant, obtained from the sum of the scores of negative dominant and positive dominant.

Submissive, obtained by adding negative submissive and positive submissive.

Positive valence, obtained by adding positive and dominant positive submissive.

Negative valence, obtained by adding negative dominant and negative submissive.

In this paper, we used the Spanish adaptation of the MiniPONS carried out by Martínez-Sánchez et al. (2013). The psychometric properties of the MiniPONS have been documented by Bänziger et al. (2011a). In Spanish population, these properties can be seen in the study of Martínez-Sánchez et al. (2013).

##### Statistical analysis

We perform the same statistical analyses as in the first study.

## Results

### Internal Consistencies and Descriptive Statistics

Cronbach alphas, means, and standard deviations were calculated for each EI scale. Table 4 presents the results for the total sample of participants, and by gender. All the internal consistency values of the EI measures were within acceptable levels. The Kuder-Richardson formula 20 (KR-20), means, and standard deviations were calculated for each channel and quadrant of the MiniPONS. As can be seen in Table 4, the values of internal consistency of the MiniPONS did not present a very acceptable level. The highest value was that of the total test (0.420), in the same line but somewhat lower than that obtained for the total test (0.566) by the authors (Bänziger et al., 2011a), and than that (0.563) of the adaptation (Martínez-Sánchez et al., 2013).

**Table 4:** Cronbach’s alphas, means, standard deviations of the variables examined.

Trait meta-mood scale (TMMS)							
	**Total sample *N* = 646**			**Male *N* = 147**		**Female *N* = 499**	
			
**Scale (number of items)**	**Cronbach alpha**	***M***	***SD***	***M***	***SD***	***M***	***SD***

Scale (number of items)	Cronbach alpha	*M*	*SD*	*M*	*SD*	*M*	*SD*
Attention (8)	0.893	27.20	6.20	25.42	6.21	27.72	6.10
Clarity (8)	0.879	28.20	6.53	29.37	7.05	27.86	6.33
Repair (8)	0.875	28.09	6.21	29.00	6.13	27.82	6.21

**Trait emotional intelligence questionnaire (TEIQue)**							

	**Total Sample *N* = 646**			**Male *N* = 147**		**Female *N* = 499**	
			
**Scale (facet) (number of items)**	**Cronbach alpha**	***M***	***SD***	***M***	***SD***	***M***	***SD***

Emotion expression (10)	0.901	4.78	1.30	4.55	1.29	4.85	1.30
Empathy (trait empathy) (9)	0.748	5.53	0.76	5.31	0.78	5.59	0.74
Self-motivation (10)	0.816	5.18	0.92	5.03	0.89	5.22	0.92
Emotion regulation (12)	0.842	4.39	0.94	4.76	0.93	4.28	0.91
Happiness (trait happiness) (8)	0.879	5.55	1.08	5.49	1.14	5.57	1.06
Social awareness (11)	0.833	4.91	0.95	4.96	0.98	4.90	0.94
Impulsiveness (low) (9)	0.776	5.04	0.93	5.13	0.95	5.02	0.93
Emotion perception (self an others) (10)	0.803	5.14	0.88	4.99	0.88	5.18	0.88
Self-esteem (11)	0.840	4.94	0.94	5.08	0.81	4.90	0.98
Assertiveness (9)	0.777	4.73	0.94	4.92	1.03	4.68	0.91
Emotion management (others) (9)	0.783	4.83	0.92	4.98	0.90	4.79	0.93
Optimism (trait optimism) (8)	0.903	5.30	1.16	5.36	1.17	5.29	1.15
Relationship (9)	0.636	5.62	0.72	5.42	0.82	5.68	0.67
Adaptability (9)	0.794	4.69	0.95	4.85	0.95	4.64	0.94
Stress management (10)	0.837	4.58	1.06	4.94	1.11	4.47	1.02
Well-being factor (3 facets)	0.860	5.26	0.94	5.31	0.93	5.25	0.94
Emotionality factor (4 facets)	0.740	5.27	0.70	5.07	0.74	5.32	0.69
Sociability factor (3 facets)	0.755	4.83	0.77	4.95	0.82	4.79	0.75
Self-control factor (3 facets)	0.829	4.67	0.84	4.95	0.86	4.59	0.82
Global trait EI (15 facets)	0.907	5.01	0.64	5.05	0.68	5.00	0.63

**Mini profile of non-verbal sensitivity (MiniPONS)**							

	**Total sample *N* = 646**			**Male *N* = 147**		**Female *N* = 499**	
			
**Channel (modality) (number of items)**	**KR-20**	***M***	***SD***	***M***	***SD***	***M***	***SD***

CF + RS (audio) (16)	0.327	12.10	1.96	12.05	1.98	12.11	1.96
Body (video) (16)	0.139	12.44	1.66	12.16	1.52	12.52	1.70
Face (video) (16)	0.082	12.64	1.45	12.32	1.45	12.73	1.43
Body + Face (video) (32)	0.210	25.07	2.34	24.48	2.21	25.25	2.35
Face & CF + Face & RS (both audio & video) (16)	0.189	13.26	1.58	12.99	1.57	13.34	1.57
Total MiniPONS (64)	0.420	50.43	3.94	49.52	4.07	50.70	3.86

**Quadrant (number of items)**	**KR-20**	**M**	**SD**	**M**	**SD**	**M**	**SD**

Negative dominant (16)	0.026	12.28	1.56	11.89	1.68	12.39	1.51
Negative submissive (16)	0.261	12.49	1.75	12.27	1.75	12.56	1.74
Positive dominant (16)	0.264	12.78	1.71	12.61	1.70	12.83	1.72
Positive submissive (16)	0.101	12.88	1.55	12.75	1.49	12.92	1.56
Dominant (32)	0.228	24.98	2.47	24.39	2.49	25.15	2.45
Submissive (32)	0.278	25.45	2.44	25.13	2.59	25.55	2.38
Positive valence (32)	0.326	25.43	2.58	25.14	2.56	25.52	2.58
Negative valence (32)	0.208	25.00	2.42	24.38	2.48	25.18	2.37


*CF = content-filtered speech; RS = randomized-spliced speech; Dominant = negative dominant + positive dominant; Submissive = negative submissive + positive submissive; Positive valence = positive dominant + positive submissive; Negative valence = negative dominant + negative submissive.*

Janusik (2017) collect the opinions of Hall (2001) which indicates that “due to low interitem correlations, the shorter forms of the PONS (Face and Body, Vocal Expression, and MiniPONS) tend to generate poor reliability estimates, and argued that the standard psychometric model might not be applicable to non-verbal sensitivity tests” Janusik (2017, p. 524).

### Correlations Between the EI Dimensions and the Channel Scores of the MiniPONS

Pearson product moment correlations were computed between the dimensions of the EI measures (TMMS, TEIQue) and the channel scores of the MiniPONS. We also examined the relation between participants’ age and the channel scores of the MiniPONS.

As shown in Table 5, not all the EI dimensions were significantly related to the channels and quadrants of the MiniPONS. Regarding the EI dimensions of the TMMS, the repair dimension did not present any significant relationship; Clarity only presented a significant and positive relationship with the score of the CF+RS channel; whereas attention presented significant and positive relations with the scores of CF+RS, body, body+face, negative submissive, positive dominant, dominant, submisive, positive valence, negative valence, and total MiniPONS.

**Table 5:** Pearson correlations between the emotional intelligence dimensions and the mini profile of non-verbal sensitivity (MiniPONS).

	Mini profile of non-verbal sensitivity (MiniPONS)													
	
	CF + RS	Body	Face	Body + Face	Face & CF + Face & RS	Total MiniPONS	Negative dominant	Negative submissive	Positive dominant	Positive submissive	Dominant	Submissive	Positive valence	Negative valence
**Trait meta-mood scale (TMMS)**													
Attention	0.119^∗∗^	0.107^∗∗^	0.060	0.114^∗∗^	0.013	0.132^∗∗∗^	0.062	0.099^∗^	0.092^∗^	0.060	0.125^∗∗∗^	0.087^∗^	0.100^∗^	0.109^∗∗^
	(*p* = 0.002)	(*p* = 0.006)	(*p* = 0.129)	(*p* = 0.004)	(*p* = 0.735)	(*p* = 0.001)	(*p* = 0.116)	(*p* = 0.012)	(*p* = 0.019)	(*p* = 0.128)	(*p* = 0.001)	(*p* = 0.027)	(*p* = 0.011)	(*p* = 0.006)
Clarity	0.078^∗^	-0.018	-0.011	-0.019	-0.020	0.019	0.041	0.028	-0.013	-0.010	0.038	-0.007	0.025	0.004
	(*p* = 0.049)	(*p* = 0.650)	(*p* = 0.788)	(*p* = 0.624)	(*p* = 0.614)	(*p* = 0.626)	(*p* = 0.294)	(*p* = 0.473)	(*p* = 0.739)	(*p* = 0.794)	(*p* = 0.338)	(*p* = 0.852)	(*p* = 0.522)	(*p* = 0.912)
Repair	-0.014	-0.038	0.028	-0.010	-0.027	-0.024	0.018	-0.002	-0.031	-0.041	0.009	-0.047	0.003	-0.042
	(*p* = 0.730)	(*p* = 0.340)	(*p* = 0.482)	(*p* = 0.807)	(*p* = 0.486)	(*p* = 0.550)	(*p* = 0.648)	(*p* = 0.962)	(*p* = 0.426)	(*p* = 0.297)	(*p* = 0.818)	(*p* = 0.230)	(*p* = 0.931)	(*p* = 0.287)
**Trait emotional intelligence questionnaire (TEIQue)**													
Well-being factor	-0.112^∗∗^	-0.058	-0.005	-0.044	-0.013	-0.087^∗^	0.040	-0.077^∗^	-0.106^∗∗^	-0.057	-0.067	-0.073	-0.079^∗^	-0.058


	(*p* = 0.004)	(*p* = 0.144)	(*p* = 0.908)	(*p* = 0.266)	(*p* = 0.736)	(*p* = 0.027)	(*p* = 0.308)	(*p* = 0.049)	(*p* = 0.007)	(*p* = 0.147)	(*p* = 0.090)	(*p* = 0.064)	(*p* = 0.045)	(*p* = 0.143)
Emotionality factor	-0.023	0.004	0.021	0.016	0.049	0.017	0.085^∗^	0.026	-0.057	-0.007	-0.001	0.028	-0.031	0.062


	(*p* = 0.560)	(*p* = 0.929)	(*p* = 0.595)	(*p* = 0.694)	(*p* = 0.217)	(*p* = 0.661)	(*p* = 0.032)	(*p* = 0.516)	(*p* = 0.148)	(*p* = 0.857)	(*p* = 0.990)	(*p* = 0.471)	(*p* = 0.424)	(*p* = 0.117)
Sociability factor	-0.097^∗^	-0.039	-0.012	-0.035	0.041	-0.053	0.021	-0.045	-0.090^∗^	-0.004	-0.053	-0.031	-0.058	-0.023


	(*p* = 0.014)	(*p* = 0.322)	(*p* = 0.770)	(*p* = 0.375)	(*p* = 0.301)	(*p* = 0.182)	(*p* = 0.602)	(*p* = 0.248)	(*p* = 0.023)	(*p* = 0.924)	(*p* = 0.182)	(*p* = 0.425)	(*p* = 0.139)	(*p* = 0.554)
Self-control factor	-0.109^∗∗^	-0.035	-0.031	-0.044	-0.033	-0.094^∗^	-0.044	-0.064	-0.058	-0.059	-0.099^∗^	-0.051	-0.042	-0.109^∗∗^


	(*p* = 0.005)	(*p* = 0.368)	(*p* = 0.436)	(*p* = 0.261)	(*p* = 0.398)	(*p* = 0.016)	(*p* = 0.265)	(*p* = 0.103)	(*p* = 0.143)	(*p* = 0.135)	(*p* = 0.011)	(*p* = 0.193)	(*p* = 0.286)	(*p* = 0.006)
Global trait EI	-0.107^∗∗^	-0.041	-0.008	-0.034	0.005	-0.072	0.039	-0.051	-0.107^∗∗^	-0.045	-0.070	-0.045	-0.072	-0.040
	(*p* = 0.006)	(*p* = 0.303)	(*p* = 0.841)	(*p* = 0.391)	(*p* = 0.899)	(*p* = 0.069)	(*p* = 0.323)	(*p* = 0.194)	(*p* = 0.006)	(*p* = 0.253)	(*p* = 0.076)	(*p* = 0.256)	(*p* = 0.067)	(*p* = 0.311)
**Age**	-0.101^∗∗^	-0.147^∗∗∗^	-0.098^∗^	-0.166^∗∗∗^	-0.101^∗∗^	-0.190^∗∗∗^	0.018	-0.119^∗∗^	-0.207^∗∗∗^	-0.137^∗∗∗^	-0.139^∗∗∗^	-0.164^∗∗∗^	-0.180^∗∗∗^	-0.117^∗∗^
	(*p* = 0.010)	(*p* = 0.000)	(*p* = 0.013)	(*p* = 0.000)	(*p* = 0.010)	(*p* = 0.000)	(*p* = 0.641)	(*p* = 0.003)	(*p* = 0.000)	(*p* = 0.000)	(*p* = 0.000)	(*p* = 0.000)	(*p* = 0.000)	(*p* = 0.003)
**Short multichannel version of the profile of non-verbal sensitivity (MiniPONS)**													
Channel (modality)													
CF + RS (audio)		0.111^∗∗^	0.139^∗∗∗^	0.165^∗∗∗^	0.148^∗∗∗^	0.656^∗∗∗^	0.286^∗∗∗^	0.384^∗∗∗^	0.485^∗∗∗^	0.408^∗∗∗^	0.515^∗∗∗^	0.536^∗∗∗^	0.581^∗∗∗^	0.449^∗∗∗^
		(*p* = 0.005)	(*p* = 0.000)	(*p* = 0.000)	(*p* = 0.000)	(*p* = 0.000)	(*p* = 0.000)	(*p* = 0.000)	(*p* = 0.000)	(*p* = 0.000)	(*p* = 0.000)	(*p* = 0.000)	(*p* = 0.000)	(*p* = 0.000)
Body (video)			0.122^∗∗^	0.788^∗∗∗^	0.105^∗∗^	0.565^∗∗∗^	0.223^∗∗∗^	0.313^∗∗∗^	0.400^∗∗∗^	0.415^∗∗∗^	0.408^∗∗∗^	0.498^∗∗∗^	0.464^∗∗∗^	0.425^∗∗∗^
			(*p* = 0.002)	(*p* = 0.000)	(*p* = 0.008)	(*p* = 0.000)	(*p* = 0.000)	(*p* = 0.000)	(*p* = 0.000)	(*p* = 0.000)	(*p* = 0.000)	(*p* = 0.000)	(*p* = 0.000)	(*p* = 0.000)
Face (video)				0.707^∗∗∗^	0.149^∗∗∗^	0.549^∗∗∗^	0.377^∗∗∗^	0.402^∗∗∗^	0.349^∗∗∗^	0.174^∗∗∗^	0.510^∗∗∗^	0.369^∗∗∗^	0.402^∗∗∗^	0.465^∗∗∗^
				(*p* = 0.000)	(*p* = 0.000)	(*p* = 0.000)	(*p* = 0.000)	(*p* = 0.000)	(*p* = 0.000)	(*p* = 0.000)	(*p* = 0.000)	(*p* = 0.000)	(*p* = 0.000)	(*p* = 0.000)
Body + Face (video)					0.167^∗∗∗^	0.743^∗∗∗^	0.393^∗∗∗^	0.472^∗∗∗^	0.501^∗∗∗^	0.404^∗∗∗^	0.607^∗∗∗^	0.583^∗∗∗^	0.580^∗∗∗^	0.592^∗∗∗^
					(*p* = 0.000)	(*p* = 0.000)	(*p* = 0.000)	(*p* = 0.000)	(*p* = 0.000)	(*p* = 0.000)	(*p* = 0.000)	(*p* = 0.000)	(*p* = 0.000)	(*p* = 0.000)
Face & CF + Face & RS (both audio & video)						0.575^∗∗∗^	0.276^∗∗∗^	0.445^∗∗∗^	0.362^∗∗∗^	0.278^∗∗∗^	0.465^∗∗∗^	0.456^∗∗∗^	0.420^∗∗∗^	0.488^∗∗∗^
						(*p* = 0.000)	(*p* = 0.000)	(*p* = 0.000)	(*p* = 0.000)	(*p* = 0.000)	(*p* = 0.000)	(*p* = 0.000)	(*p* = 0.000)	(*p* = 0.000)
Total MiniPONS							0.487^∗∗∗^	0.651^∗∗∗^	0.686^∗∗∗^	0.555^∗∗∗^	0.805^∗∗∗^	0.797^∗∗∗^	0.803^∗∗∗^	0.771^∗∗∗^
							(*p* = 0.000)	(*p* = 0.000)	(*p* = 0.000)	(*p* = 0.000)	(*p* = 0.000)	(*p* = 0.000)	(*p* = 0.000)	(*p* = 0.000)
Quadrant													
Negative dominant								0.068	0.096^∗^	0.045	0.677^∗∗∗^	0.098^∗^	0.348^∗∗∗^	0.421^∗∗∗^
								(*p* = 0.084)	(*p* = 0.015)	(*p* = 0.251)	(*p* = 0.000)	(*p* = 0.012)	(*p* = 0.000)	(*p* = 0.000)
Negative submissive									0.297^∗∗∗^	0.125^∗∗∗^	0.489^∗∗∗^	0.554^∗∗∗^	0.290^∗∗∗^	0.750^∗∗∗^
									(*p* = 0.000)	(*p* = 0.001)	(*p* = 0.000)	(*p* = 0.000)	(*p* = 0.000)	(*p* = 0.000)
Positive dominant										0.202^∗∗∗^	0.597^∗∗∗^	0.501^∗∗∗^	0.808^∗∗∗^	0.254^∗∗∗^
										(*p* = 0.000)	(*p* = 0.000)	(*p* = 0.000)	(*p* = 0.000)	(*p* = 0.000)
Positive submissive											0.148^∗∗∗^	0.746^∗∗∗^	0.467^∗∗∗^	0.406^∗∗∗^
											(*p* = 0.000)	(*p* = 0.000)	(*p* = 0.000)	(*p* = 0.000)
Dominant												0.283^∗∗∗^	0.667^∗∗∗^	0.598^∗∗∗^
												(*p* = 0.000)	(*p* = 0.000)	(*p* = 0.000)
Submissive													0.618^∗∗∗^	0.638^∗∗∗^
													(*p* = 0.000)	(*p* = 0.000)
Positive valence														0.240^∗∗∗^
														(*p* = 0.000)


*CF = content-filtered speech; RS = randomized-spliced speech; Dominant = negative dominant + positive dominant; Submissive = negative submissive + positive submissive; Positive valence = positive dominant + positive submissive; Negative valence = negative dominant + negative submissive; Total sample *N* = 646. ^∗∗∗^*p* ≤ 0.001 (two tailed); ^∗∗^*p* ≤ 0.01 (two tailed); ^∗^*p* ≤ 0.05 (two tailed).*

Of the EI factors of the TeiQUE, the following relationships were significant: the well-being factor presented a significant and negative relationship with CF+RS, negative submissive, positive dominant, positive valence, and the total MiniPONS. The emotionality factor only presented a significant and positive relationship with negative dominant. The sociability factor presented significant and negative relationships with CF+RS and positive dominant. The self-control factor presented significant and negative relations with CF+RS, dominant, negative valence, and the total MiniPONS.

Age presented significant and negative relationship with all the channels and quadrants except for the negative dominant.

### Stepwise Multiple Regression With EI Dimensions as Predictor Variables and the Channel Scores of the MiniPONS as Criterial Variables

Prior to the stepwise multiple regression analyses, the relationships between independent variables (TMMS, TEIQue) and the dependent variables (MiniPONS) were examined. EI dimensions variables significantly associated with channel scores variables were considered candidate predictors and were entered into the stepwise multiple regression analysis.

Age was considered as an independent variable in the situations in which it had a significant relation with some scores of the MiniPONS.

Gender was entered as an independent variable to determine whether it predicted non-verbal sensitivity only in cases where significant differences between men and women were found, that is, in the following channels: Body *t*(644) = -2.268, *p* = 0.024, *d* = -0.223; Face *t*(644) = -3.044, *p* = 0.002, *d* = -0.283; Body+Face *t*(644) = -3.517*, p* = 0.000, *d* = -0.337; Face & CF+Face & RS *t*(644) = -2.379*, p* = 0.018, *d* = -0.222; and total MiniPONS *t*(644) = -3.215*, p* = 0.001, *d* = -0.297. In all of them, women’s scores were higher than men’s (see Table 4). We also found significant differences between men and women in the scores of the quadrants negative dominant *t*(644) = -3.446*, p* = 0.001, *d* = -0.313; dominant *t*(644) = -3.312, *p* = 0.001, *d* = -0.307; and negative valence *t*(644) = -3.534*, p* = 0.000, *d* = -0.329. In all of them, women’s scores were higher than men’s.

The results are presented in Table 6. In general, very few EI dimensions predicted Non-verbal Sensitivity, and the same prediction results did not appear in all the channels and scores of the MiniPONS quadrants.

**Table 6:** Stepwise multiple regression analysis.

Dependent variables: Components of the mini profile of non-verbal sensitivity (MiniPONS)										
Independent variables: Dimensions of the trait meta-mood scale (TMMS) and trait emotional intelligence questionnaire (TEIQue)										
**Model**	***R***	***R*^2^**	***R*^2^ adjusted**	***R*^2^ change**	***F*(df)**	***p***	**ß**	**ß standarized**	***t***	***p***

**Dependent variable: CF + RS (audio)**										
Model 1: Attention	0.119	0.014	0.013	0.014	9.285 (1,644)^∗∗^	0.002	0.038	0.119	3.047^**^	0.002
Model 2: Attention							0.037	0.116	2.977^**^	0.003
Sociability factor	0.151	0.023	0.020	0.009	7.501 (2,643)^∗∗∗^	0.001	-0.236	-0.093	-2.377^*^	0.018
Model 3: Attention							0.029	0.093	2.317^*^	0.021
Sociability factor							-0.337	-0.132	-3.151^**^	0.002
Clarity	0.179	0.032	0.028	0.009	7.095 (3,642)^∗∗∗^	0.000	0.032	0.107	2.483^*^	0.013
Model 4: Attention							0.024	0.074	1.817	0.070
Sociability factor							-0.325	-0.127	-3.036^**^	0.002
Clarity							0.034	0.114	2.660^**^	0.008
Age	0.195	0.038	0.032	0.006	6.365 (4,641)^∗∗∗^	0.000	-0.017	-0.081	-2.018^*^	0.044
**Dependent variable: Negative dominant**										
Model 1: Gender	0.135	0.018	0.017	0.018	11.877 (1,644)^∗∗∗^	0.001	0.502	0.135	3.446^***^	0.001
**Dependent variable: Negative submissive**										
Model 1: Age	0.119	0.014	0.013	0.014	9.205 (1,644)^∗∗^	0.003	-0.022	-0.119	-3.034^**^	0.003
**Dependent variable: Positive dominant**										
Model 1: Age	0.207	0.043	0.041	0.043	28.848 (1,644)^∗∗∗^	0.000	-0.037	-0.207	-5.371^***^	0.000
**Dependent variable: Dominant**										
Model 1: Age	0.139	0.019	0.018	0.019	12.777 (1,644)^∗∗∗^	0.000	-0.036	-0.139	-3.574^***^	0.000
Model 2: Age							-0.033	-0.127	-3.252^***^	0.001
Gender	0.181	0.033	0.030	0.013	10.852 (2,643)^∗∗∗^	0.000	0.683	0.116	2.962^**^	0.003
Model 3: Age							-0.029	-0.110	-2.778^**^	0.006
Gender							0.615	0.104	2.649^**^	0.0008
Attention	0.199	0.040	0.035	0.007	8.806 (3,642)^∗∗∗^	0.000	0.034	0.086	2.143^*^	0.032
**Dependent variable: Positive valence**										
Model 1: Age	0.180	0.032	0.031	0.032	21.570 (1,644)^∗∗∗^	0.000	-0.049	-0.180	-4.644^***^	0.000
**Dependent variable: Negative valence**										
Model 1: Gender	0.138	0.019	0.017	0.019	12.486 (1,644)^∗∗∗^	0.000	0.795	0.138	3.534^***^	0.000
Model 2: Gender							0.731	0.127	3.242^***^	0.001
Age	0.172	0.029	0.026	0.010	9.756 (2,643)^∗∗∗^	0.000	-0.026	-0.103	-2.629^**^	0.009
**Dependent variable: Total MiniPONS**										
Model 1: Age	0.190	0.036	0.034	0.036	24.010 (1,644)^∗∗∗^	0.000	-0.078	-0.190	-4.900^***^	0.000
Model 2: Age							-0.074	-0.178	-4.596^***^	0.000
Gender	0.217	0.047	0.044	0.011	15.894 (2,643)^∗∗∗^	0.000	0.998	0.106	2.745^**^	0.006
Model 3: Age							-0.067	-0.162	-4.106^***^	0.000
Gender							0.892	0.095	2.437^*^	0.015
Attention	0.232	0.054	0.049	0.007	12.140 (3,642)^∗∗∗^	0.000	0.053	0.084	2.112^*^	0.035


*CF = content-filtered speech; RS = randomized-spliced speech; Dominant = negative dominant + positive dominant; Submissive = negative submissive + positive submissive; Positive valence = positive dominant + positive submissive; Negative valence = negative dominant + negative submissive; Total sample *N* = 646. ^∗∗∗^*p* ≤ 0.001 (two tailed); ^∗∗^*p* ≤ 0.01 (two tailed); ^∗^*p* ≤ 0.05 (two tailed).*

In the CF+RS channel, the prediction model contained four predictors and was reached in four steps, *F*(4,641) = 6.365, *p* < 0.001, accounting for 3.8% of the variance of the CF+RS channel (*R*^2^ = 0.038). The significant predictors of this model were attention (*R*^2^ = 0.014), the Sociability Factor (*R*^2^ = 0.009; with a negative relation, see Table 5), clarity (*R*^2^ = 0.009), and age (*R*^2^ = 0.006; with a negative relation, see Table 5). attention was the EI dimension that best predicted the CF+RS channel.

In the total MiniPONS, the prediction model contained three predictors and was reached in three steps, *F*(3,642) = 12.140, *p* < 0.001, accounting for 5.4% of the variance of the total MiniPONS (*R*^2^ = 0.054). The significant predictors of this model were age (*R*^2^ = 0.036; with a negative relation, see Table 5), gender (*R*^2^ = 0.011; with women obtaining higher scores than men, see Table 4), and attention (*R*^2^ = 0.007).

In the negative dominant quadrant, the prediction model contained one predictor and was reached in one step, *F*(1,644) = 11.877*, p* = 0.001, accounting for 1.8% of the variance of the negative dominant quadrant (*R*^2^ = 0.018). The significant predictors of this model were gender (*R*^2^ = 0.018; with women obtaining higher scores than men).

In the negative submissive quadrant, the prediction model contained one predictor and was reached in one step, *F*(1,644) = 9.205*, p* = 0.003, accounting for 1.4% of the variance of the negative submissive quadrant (*R*^2^ = 0.014). The significant predictor of this model was age (*R*^2^ = 0.014; with a negative relation, see Table 5).

Similarly to the previous quadrant, in the positive dominant quadrant, the prediction model contained one predictor and was reached in one step, *F*(1,644) = 28.848, *p* < 0.001, accounting for 4.3% of the variance of the positive dominant quadrant (*R*^2^ = 0.043). The significant predictor of this model was age (*R*^2^ = 0.043; with a negative relation, see Table 5).

In the dominant quadrant, the prediction model contained three predictors and was reached in three steps, *F*(3,642) = 8.806, *p* < 0.001, accounting for 4.0% of the variance of the dominant quadrant (*R*^2^ = 0.040). The significant predictors of this model were age (*R*^2^ = 0.019; with a negative relation, see Table 5), gender (*R*^2^ = 0.013; with women obtaining higher scores than men, see Table 4), and attention (R^2^ = 0.007). In this quadrant and the total MiniPONS, the same predictors emerged.

In the positive valence quadrant, the prediction model contained one predictor and was reached in one step, *F*(1,644) = 21.570, *p* < 0.001, accounting for 3.2% of the variance of the positive valence quadrant (*R*^2^ = 0.032). The significant predictors of this model was Age (*R*^2^ = 0.032; with a negative relation, see Table 5).

Finally, in the negative valence quadrant, the prediction model contained two predictors and was reached in two steps, *F*(2,643) = 9.756, *p* < 0.001, accounting for 2.9% of the variance of the negative valence quadrant (*R*^2^ = 0.029). The significant predictors of this model were gender (*R*^2^ = 0.019; with women obtaining higher scores than men, see Table 4), and age (*R*^2^ = 0.010; with a negative relation, see Table 5).

Results showed that the only EI dimensions that predicted non-verbal sensitivity were attention, clarity, and the sociability factor [which includes the facets of social awareness, emotion management (others), and Assertiveness]. Of these three EI dimensions, the one that best predicted non-verbal sensitivity was attention. However, the prediction was generally not very strong.

## Conclusion

The purpose of this study was to examine the relationship between EI and empathy, on the one hand, and, on the other, the relationship between EI and non-verbal sensitivity, through two independent studies.

The investigations carried out to date have usually applied a single EI measure to verify the relation between EI and empathy. One of our goals was to obtain results with the highest possible number of EI dimensions to determine those that best predict empathy, so we applied the two measures that are most commonly used, assessing different domains.

The descriptive statistics and the internal consistency of the EI measurements, in both studies, are in line with those obtained by us in a study on EI and health, where we apply the same scales of EI in a similar population (Fernández-Abascal and Martín-Díaz, 2015).

With respect to the proposed objectives, in the first study, the results show a similar pattern of significant positive relationships of all the analyzed EI domains with empathic PT, and significant negative relationships with empathic PD, except for the EI domain attention, which presents a positive relationship with empathic PD. The negative relationships established between all the domains of EI, with the exception of the domain of attention, and the empathy factor PD are in the line of our expectations because PD is a negative social factor that reflects a person’s feelings of anxiety and discomfort when observing other people’s negative experiences.

With the other two aspects of empathy, fantasy and EC, their relations with the EI domains were not homogeneous, and in some domains, no significant relationships were established.

Regarding the results of the specific EI dimensions that best predict aspects of empathy, the data showed that not all EI dimensions have the same predictive power for empathy.

For the two cognitive empathy scales, PT and fantasy, the results showed that the EI dimension that best predicts empathic PT is the Emotionality Factor, which reflects self efficacy in perceiving and expressing emotions and subsequently using them to create and maintain relationships with others, and it includes the facets of emotion perception (self and others), emotion expression, Trait empathy, and relationships. In addition to other facets, the facet of empathy is contemplated within this factor, and this circumstance cannot be ignored, the construct of EI includes components of empathy.

The EI dimension that best predicts empathic fantasy is attention, which reflects the degree to which people tend to observe and think about their feelings and moods.

For the two emotional empathy scales, EC and PD, the EI dimension that best predicts EC is the emotionality factor, as with empathic PT. The self-control factor [reflecting self-efficacy in emoton/impulse regulation, and including the facets of emotion regulation, Stress Management, and (low) Impulsiveness] is the EI dimension that best predicts empathic PD, but the relationship between them is negative, as can be seen in the analysis of the correlations.

Alterman et al. (2003) pointed out that the most robust components of empathy seem to be represented in the scales of PT and EC and, in our study, the emotionality factor is the common predictor of both component.

Gender emerges as a predictor in three of the four analyzed aspects of empathy, fantasy, EC, and PD, and in all of them, women score higher than men. We had previously found significant gender differences in these three aspects of empathy, and PT was the only aspect where no differences were found.

Age is only a predictor of empathic fantasy, and the relationship is negative. However, age also presented significant negative relationships with PD: as age increases, the score in both these aspects decreases.

We cannot compare these results with those obtained in other studies, as we have not found any research that uses so many EI domains as predictors. However, some results can be partially compared with those obtained by Extremera and Fernández-Berrocal (2004) because, in their research, they used two of the measures employed in our first study, the TMMS and the IRI. In their analysis of the relationships between the two tests, they found correlations pointing in the same direction as those of our study, although they found no positive relationships between the clarity and repair dimensions and EC, as we did in our work. These authors also studied the contribution of EI assessed with TMMS as a predictor of empathy assessed with IRI, finding fewer predictive EI dimensions than ours, and moreover, they found no EI dimension predicting empathic fantasy. However, in our study, the attention and clarity dimensions were entered in the prediction model, and clarity was the dimension that best predicted fantasy.

The differences observed in men’s and women’s scores in empathy, with women obtaining higher scores than men, agree with some of those obtained in studies of adaptation of the test to other languages; for example, Pérez-Albéniz et al. (2003) found differences in the same aspects as we found in our study; Gilet et al. (2013) found differences in the aspects of fantasy and EC, and De Corte et al. (2007) found differences in all the aspects. It seems that, in general, women score higher on measures of empathy than men (e.g., Wright and Skagerberg, 2012; Clarke et al., 2016).

The results concerning age, with younger people scoring higher, are similar to those obtained, for example, by Gilet et al. (2013) in the same empathic aspects as in our study, fantasy and PD. Davis (1983) emphasized that “PD is an egocentric precursor of more mature empathy and that it decreases with age and emotional maturity” (Young Kaelber and Schwartz, 2014, p. 279).

Regarding the second study, which examined the relationship between EI and non-verbal sensitivity, like the first study, the most usual solution has been to apply a single measure of EI to verify this relationship. However, because one of our goals was to obtain results with the highest number of EI dimensions in order to determine those that best predict non-verbal sensitivity, we also applied the two measures that we had administered in the first study.

The results do not show many relations between the two constructs; notably, attention is the EI dimension that presents more significant relationships – all of them positive – with CF+RS, body, body+face, negative submissive, positive dominant, dominant, submisive, positive valence, negative valence, and Total MiniPONS. Another relevant result is the significant negative relationships between three EI factors, well-being, sociability, and self-control, with some channels and quadrants of MiniPONS. The emotionality factor only presents a significant and positive relationship with the quadrant Negative dominant.

Very few EI dimensions predict non-verbal sensitivity, and there were no predictive results in some of the channels and quadrants of the MiniPONS and, furthermore, they had little predictive value.

The highest number of EI dimensions that are predictors emerge in channel CF+RS: attention, the sociability factor (with a negative relationship), clarity, and age (with a negative relationship), but the dimension that best predicts is attention.

In the total MiniPONS the strongest predictor is age (with a negative relation): as participants’ get older, the results in non-verbal sensitivity are worse. This predictor is followed by gender: women score higher than men, and within the EI dimensions, only the dimension of attention emerges as a predictor, with a rather low value.

In the dominant quadrant, the same predictors emerge as for the total MiniPONS score.

Age emerges as the only predictor (with a negative relationship), in the negative submissive, positive dominant, and positive valence quadrants: as people age, they obtain worse results in these quadrants of non-verbal sensitivity.

Gender emerges as the only predictor in the negative dominant quadrant, with women obtaining higher scores than men.

Finally, age (with a negative relationship) and gender predict the negative valence, both of them in the same line as the results obtained in the previous channels and quadrants: as people age, they obtain worse results, and compared to men, women obtain better results.

In the analysis of the relationships between age and the channels and quadrants, age showed significant and negative relations with all of them except for negative dominant.

We found significant differences between men and women in body, face; body+face; face & CF+face & RS, and the total MiniPONS channels and in the negative dominant, dominant, and negative valence quadrants. In all of them, women obtained higher scores than men.

There are almost no studies concerning the connection between EI and non-verbal sensitivity, but we can partially compare some of the results obtained in this work, for example, with the work of Martínez-Sánchez et al. (2013), who obtained a result similar to the one of our study with the domain of attention, finding positive, and significant relationships of attention with multiple channels and quadrants of the MiniPONS, including the total test score.

The differences observed in the scores between men and women are consistent with the results obtained in other studies, showing that women obtain higher scores than men in the PONS, and the MiniPONS (Hall, 1978, 1984, Martínez-Sánchez et al., 2013; Gulabovska and Leeson, 2014). Knapp and Hall (2002) pointed out that, compared to men, women not only decode more efficiently, but they also encode non-verbal emotional cues more efficiently.

The negative correlations obtained between age and the level of non-verbal sensitivity are in line with the results found in other studies reporting that the ability to recognize facial expressions decreases with age (Mill et al., 2009), and the capacity to recognize emotional prosody also appears to be negatively affected by age (Orbelo et al., 2005; Ruffman et al., 2008).

Riggio and Darioly (2016) pointed out that, despite the obviousness of the connection between non-verbal sensitivity and EI, this connection is not well-known among researchers, apart from those who are investigating non-verbal communication. Among the reasons, they note are the time it takes to apply measures of non-verbal sensitivity, the lack of strong validity, and the concern that the readily available and better investigated measures have a fairly limited approach. These authors also state that, given the popularity of the construct of EI, the merging of these two worlds would make sense.

The results of the two studies help us to better understand the relationship between the constructs of EI and empathy, on the one hand, and EI and non-verbal sensitivity, on the other, especially with regard to the contribution of the EI dimensions as predictors of empathy and non-verbal sensitivity.

Several limitations of these two studies should be mentioned. In both of them, we applied self-report measures, so it is likely that social desirability influenced the response to the tests. On another hand, due to the cross-sectional design of the studies, the assumption of causality should be considered with caution. A longitudinal follow-up study would be valuable to address this limitation, so further research is needed using prospective designs to confirm our findings. Another limitation is the cultural homogeneity of the participants in the studies, which advises caution in the generalization of the results.

Despite these limitations, this study provides evidence of some EI dimensions that could explain some specific aspects of empathy and non-verbal sensitivity. These findings may help in future research to continue defining the specific domains of EI that contribute to non-verbal sensitivity and empathy.

The most relevant results of the two studies are those obtained in the first one, which delimits the specific domains of EI that predict each of the aspects of empathy considered in the study, such as the emotionality factor, the self-control factor, and attention. This information may prove useful to design intervention programs focused on improving empathic capacities, thereby influencing the training of specific domains of EI that better predict empathy, such as those that have emerged in this work.

## Ethics Statement

This study followed the Declaration of Helsinki and ethical guidelines.

## Author Contributions

The two authors listed have made a substantial, direct and intellectual contribution to the work, contribution to the work, conceptualization, data collection, write-up, data analysis, and approved it for publication.

## Conflict of Interest Statement

The authors declare that the research was conducted in the absence of any commercial or financial relationships that could be construed as a potential conflict of interest. The handling Editor declared a shared affiliation, though no other collaboration, with the authors at the time of review.
